# Determination of Natural Blood Plasma Melatonin Concentration of Tsigai Ewes Characteristic for Gestation and Early Postpartum Period Between Autumnal Equinox and Winter Solstice

**DOI:** 10.3390/vetsci12040336

**Published:** 2025-04-05

**Authors:** András Gáspárdy, László Gulyás, Ida Polland, Alán Alpár, Sándor György Fekete, Levente Harmat

**Affiliations:** 1 Institute for Animal Breeding, Nutrition and Laboratory Animal Science, University of Veterinary Medicine Budapest, 1078 Budapest, Hungary; sandorgyorgyfekete@gmail.com; 2 Department of Animal Sciences, Faculty of Agriculture and Food Sciences, Széchenyi István University, 9200 Mosonmagyaróvár, Hungary; gulyas.laszlo@sze.hu; 3 Veterinær Ida Marie Polland AS, 3330 Skotselv, Norway; idamarie-polland@hotmail.com; 4 Department of Anatomy, Histology and Embryology, Semmelweis University, 1094 Budapest, Hungary; alpar.alan@semmelweis.hu; 5 SE NAP Research Group of Experimental Neuroanatomy and Developmental Biology, Semmelweis University, 1094 Budapest, Hungary; 6 Experimental Farm, University of Veterinary Medicine Budapest, 2225 Üllő, Hungary; harmat.levente@univet.hu

**Keywords:** gestation age, circannual rhythm, circadian rhythm, winter solstice, melatonin, postpartum period

## Abstract

The aim of this investigation was to measure the natural nocturnal plasma melatonin concentration in gestating and fresh ewes. Studies in humans showed that maternal melatonin had a significant increase as pregnancy progressed and then decreased after birth. Two studies conducted in sheep so far, considering the entire gestation, have led to conflicting results. The breed of 16 pregnant ewes selected for the research was the Tsigai. Blood samples were taken into EDTA vacutainers predetermined times a night at different stages of their gestation. The RIA method was used to determine the melatonin concentrations. For estimation of its variations during gestation, population genetic statistics was applied. It was found that the average plasma melatonin concentration of 134 pg mL^−1^ is characteristic for the investigated period, and that it rises between the autumnal equinox and the winter solstice. Secondly, it was revealed that the average melatonin concentration adjusted for midnight is 162.4 pg mL^−1^, and its moderate variation is characteristic for the night. The investigation showed that there is no connection between the plasma melatonin concentration of the ewes and their gestational age in the Tsigai breed in Middle Europe. Our result is consistent with the results of single studies in sheep and donkey, in contrast to human observations. With regard to the nocturnal plasma melatonin, the concentration is reduced at the same level (30 pg mL^−1^) in ewes and lambs during the early postpartum period without nightly fluctuation. The expelled placenta, the constant vigilance between the mother and her lamb, and the opposition between melatonin and prolactin may provide a plausible explanation for this.

## Simple Summary

Melatonin is a special hormone with many functions. Its production in human and animal organisms is particularly seasonal and related to physiological processes. This study monitors blood plasma melatonin concentration in sheep during the whole gestation and early post-partum period, taking into account the effect of the season that has been less studied so far and in certain details for the first time. It proves that nocturnal plasma melatonin concentration in pregnant ewes increases between the autumnal equinox and the winter solstice in Central Europe in the Northern Hemisphere. It observes that nocturnal plasma melatonin concentration (between 18:00 p.m. and 06:00 a.m.) in pregnant ewes follows a less pronounced variation. Furthermore, it provides proof that nocturnal plasma melatonin concentration in pregnant ewes does not change as pregnancy progresses. It is the first to report that nocturnal plasma melatonin concentration decreases to the same level in ewes and new-born lambs immediately after birth, without nighty fluctuations.

## 1. Introduction

Melatonin is an endogenous hormone produced by the pineal gland in the brain and is secreted at night in vertebrates [1]. Melatonin is responsible for various biological functions such as the circadian rhythm [2,3]. It is a versatile hormone, commonly known simply as a regulator of the sleep cycle of animals, although it is also involved in other bodily processes, such as pubertal development and adaptation to different seasons, including the breeding season and the function and timing of the reproductive organs [4,5].

Tryptophan, necessary for the production of serotonin and hence melatonin, is an essential amino acid that sheep do not naturally have in their bodies [6], but which they get through their diets as they graze, consuming various types of grass and plants [7].

Melatonin transmits information about the photoperiod to the endocrine system. Sheep are seasonally polyestrous animals, also referred to as short day breeders. The photoperiod limits the sexual activity in sheep [8,9]. This means that their estrous cycle will occur only during a certain season of the year, and thus melatonin plays an important role in the reproductive functions of sheep [10,11]. However, in tropical and sub-tropical areas, depending on whether or not there is plentiful access to good-quality fodder, the ewes can be both aseasonal or intermittently polyestrous [12].

Melatonin also plays an important role in fighting reactive oxygen species (ROS), working effectively to neutralize free radical scavengers, which is why it may be called an indirect antioxidant. Its antioxidant function supports the reproductive system acting on the gonads, reducing oxidative stress caused by ROS [13]. Melatonin positively affects the fertilization process from the follicle development and ovulation all the way through to maternal recognition by contributing to a protection of embryo and fetus from the moment of conception. It may also help prevent a corpus luteum lysis and lead the ewe to a healthy continuation of this state until parturition [14]. The way melatonin acts is by binding itself to the MT1 and MT2 receptors in the reproductive system [15], more specifically in the ovine endometrium [16]. The fact that melatonin receptors are present in the ovine blastocysts is an indication of melatonin influencing placental development [17].

In animal husbandry, various methods of melatonin preparations have been used extensively to increase the fertility in sheep [18,19]. Exogenous melatonin implants are commonly used to increase the reproductive performance, a higher number of surviving embryos coupled with fewer abortions, and to diminish the heat stress in sheep bred out of season [20,21]. The melatonin level in ewes with melatonin implants has already been subject to earlier investigations [22,23].

Studies performed in humans showed that maternal melatonin had a significant increase as pregnancy progressed and then decreased after parturition and when the placenta was removed [24,25]. In contrast, a single early study conducted in ewes found that there is no change in melatonin production during the state of pregnancy [26].

Therefore, the aim of this investigation was to measure, through continuous monitoring of the natural nocturnal plasma melatonin concentration characteristic to the gestation period in ewes between autumnal equinox and winter solstice. With this investigation, we want to check which of two opposite phenomena is relevant in the case of sheep. We also intend to determine nocturnal melatonin concentration in mothers and their lambs immediately after birth, as data on this have not yet been published.

## 2. Materials and Methods

### 2.1. Study Design

Sixteen pregnant ewes of the Experimental Farm’s flock of Tsigai breed participated in this study. The Experimental Farm belongs to the University of Veterinary Medicine Budapest and is located at Üllő (47° N latitude and 19° E longitude). The sheep breed on which we conducted this study was the Tsigai. The Tsigai is an old, independent, long-tailed sheep breed originating in Asia Minor. During the 18th and 19th centuries, the golden age of the breed, it was unrivaled in the three primary uses of sheep: as a source of wool, milk, and mutton. After the end of 19th century, significant breeding programs diminished, and the breed lost its growing ascendancy in Hungary and became a heritage breed [27].

The project was approved as an experiment on farm animals, and it was given its own license number, which was PE/EA/01444-6/2022.

The selected individuals lived in the flock, which was weaned uniformly on 15 April. Prior to the experiment, the dams grazed on medium-quality pasture. Their body weight ranged between 53 and 57 kg, and their body condition score was generally 3.0 (on the scale of 1 to 5) at the beginning of the experiment. The 16 ewes had an average age of 4.9 years (with birth years between 2015 and 2020).

Starting from 1 June 2023, a darkening-light program began. The farm’s stock of sheep (35 adult females) spent decreasing time in the pasture and increasing barn time. A breeding ram was allowed in with the females on the starting day (1 June), so the breeding was performed via a harem-mating method. On this day, the number of natural daylight hours was 15 h and 42 min (sunrise at 4:50 a.m. and sunset at 20:32). This was gradually reduced throughout June to the natural number of hours typical for the beginning of September: 13 h and 25 min (sunrise at 6:03 a.m. and sunset at 19:28 on 1 September). The 1 September conditions of daylight were maintained for July and August.

The calculated energy and protein requirements of a ewe were 8–10 MJ NE_m_ and 110–133 g crude protein (June–August, respectively). The estimated individual meadow grass intake was 3–3.5 kg. As a supplement, the animals received 50 g of oats and 50 g of rye plus 500 g of alfalfa hay on an individual and daily basis from 1 June. Access to drinking water and licking salt were provided to them ad libitum. Wheat straw was placed under them as a litter material and was regularly updated. Daily access to pasture was maintained throughout the entire experimental period.

From 1 September, however, the animals were exposed to the natural conditions of daylight and night, which means that the daily life of the ewes followed the natural day-night rhythm from this point, starting the grazing at dawn and finished at dusk. From this time, the animals received 500 g of grain feed and 1 kg of alfalfa hay. The daily intake covered the requirements (12–13 MJ NE_m_ and 290–300 g crude protein; September–December).

The study design is based on temporal parallelism, thus overlapping the period of gestation and the period remaining until the winter solstice. The research was set up so that gestation coincided with the decline in natural light, and on the other hand, lambing occurred with successive shift until the winter solstice (Figure S1). Blood was collected five times over five nights (between 6:00 p.m. and 6:00 a.m. every 40 min or so) from the same sheep. Between September 2023 and December 2023, blood sampling took place on the following predetermined dates, falling between the autumnal equinox and the winter solstice: 29 September (6 days after the autumnal equinox), 27 October, 17 November, 29 November, and, finally, 9 December 2023 (13 days before the 2023 winter solstice). The length of daylight on the beginning of December was 8 h and 45 min (sunrise at 07:09 a.m. and sunset at 15:54 on 1 December). Samples were taken consequently from the left jugular vein.

During the first two sampling events, we took samples from all the ewes and performed a pregnancy test at the same time. An ultrasound apparatus (Animal Profi 2, Technology, Sząbruk, Poland) was used to determine the gestation of the animals. Only those mothers with a certified pregnancy status so far remained for the subsequent samplings.

Samples from 16 first-confirmed pregnant ewes were used to determine plasma melatonin concentrations during gestation. Due to the different times of conception, the ewes were at different stages of pregnancy and lambing during the study period. This is a necessary prerequisite for distinguishing the effect of gestational days from the effect of the days of the year. A total of 12 dams gave birth, so 12 dam–progeny pairs were sampled after lambing before the winter solstice.

The animals were kept in continuous darkness during night-time sampling. In each case, the sampling was preceded by a control measurement of the light intensity of the applied infrared bulb with a digital luminometer (Testo 540, Testo Saveris GmbH., Titisee-Neustadt, Germany), and the luminance was always below 5 lux.

### 2.2. Sampling and Determination of the Melatonin Concentration

First, a small area on the sheep’s neck was shorn, and around 8 mL of blood from the jugular vein were collected into EDTA vacutainers (Premium Vacuette^®^ K3E K3EDTA Blood Collection Tube Ref: 454086, Greiner Bio-One GmbH, Kremsmünster, Austria). The blood samples were stored in a cooling box at a temperature below 5 °C. On the same day, these were centrifuged using a universal centrifuge (Z 326 K, HERMLE Labortechnik GmbH, Wehingen, Germany) at 4000 rpm for 10 min at +4 °C. After centrifugation, the samples were collected into Eppendorf tubes, upon which plasma was immediately frozen and stored at −18 °C before being delivered for RIA analysis.

Melatonin concentration was determined as pg mL^−1^. For the reaction to ascertain the concentration of melatonin, following the protocol instructions, a Tecan RE29301 RIA kit (RE29301, IBL International GmbH, Hamburg, Germany) containing an anti-melatonin antibody labeled with iodine-125 (^125^I) radioisotope was used (indirect radioimmunoassay). The kit’s sensitivity was 0.9 pg mL^−1^; the intra-assay and inter-assay detection limits of the analysis were 28.8–266 and 3.5–281 pg mL^−1^, respectively, with the corresponding coefficients of variation of 3.9–6.8 and 6.2–16%, respectively. Melatonin concentrations were measured using a gamma counter (Perkin Elmer Wallac Wizard 1470, Wallac Oy, Turku, Finland). Samples (200 µL) were measured in duplicate, and their average was calculated, which was considered as the raw sample value (Figure S2).

### 2.3. Statistical Processing

The number of days from ram introduction to conception was calculated from the date of parturition. The gestation period was assumed to be 150 days [28]. A survival analysis (Kaplan–Meier method) was performed to determine the cumulative pregnancy rate.

The following details were recorded and calculated as background data for the processing of the melatonin results:

- Identification number: ear tag with life number;
- Sampling date: the same day that the blood was collected;
- Sampling time: the exact time when the blood was collected;
- Days to winter solstice: the number of days between the sampling date and 21 December 2023 (ranging between −83 and −13 days);
- Minutes around midnight: the number of minutes between midnight and the exact time of the blood collection (ranging between −345 and 323 min);
- Lambing date: the day when the parturition was to occur;
- Days to lambing: the number of days between the date of sampling and the date of lambing (ranging between −138 and −2);
- Days from lambing: the number of days between the date of lambing and the date of sampling (ranging between 0 and 19).

In two steps, we started the statistical processing of the obtained melatonin concentration.

In the first step, the raw sample values were corrected by eliminating the influencing effect of the sampling day (days to winter solstice) and minute (minutes around midnight) as well as the gestational age (the inverse of the days to lambing). To do this, an individual animal model of the software Pedigree Viewer version 6.5f [29] was used. During the processing, the effect on which the course of melatonin concentration was actually investigated was removed from the model.

In the second step, the adjusted values for different specific times (e.g., midnight at autumnal equinox, afternoon 6:00 p.m. at mid period, and midnight on day 98 before lambing and day one after lambing) were estimated. For comparison of the adjusted values, a statistical test (one-way ANOVA with Tukey honesty significant difference test) was performed. As a result, the mean and SEM (Standard Error of Mean) were displayed. For the calculation of regression, differences, and graphical presentation, the software Statistica (version 14., TIBCO Software Inc., Palo Alto, CA, USA) [30] was applied.

## 3. Results

### 3.1. Conception Results

**Figure 1:**
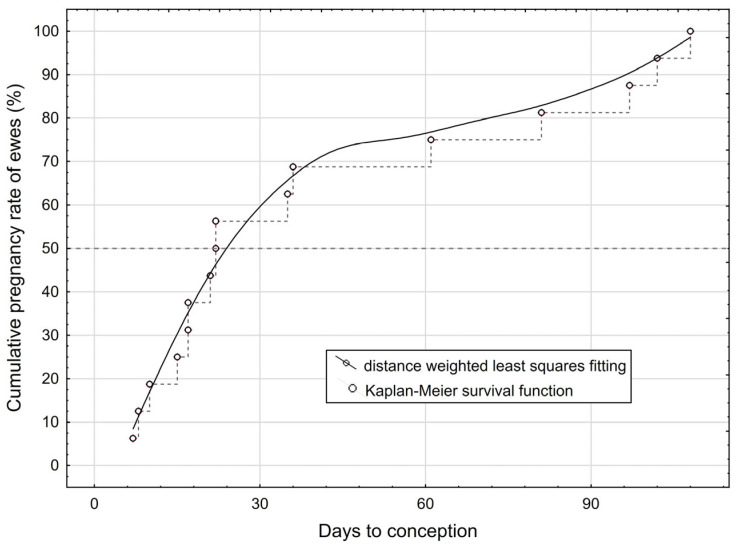
Distribution of cumulative pregnancy rate of ewes.

The estimated average number of days from opening the harem to conception for the 16 pregnant ewes was 41.2 (minimum 7 and maximum 108 days). Figure 1 illustrates that 50% of mothers became pregnant by day 22 (median) and almost 75% within the first two months. Twelve of the mothers gave birth during the study period and four (with a days to conception of more than 80 days) afterwards. Of the 12 lambings, three were twin births (two ram twin pairs and one mixed-sex twin pair). A total of 15 lambs were born (9 ram lambs and 6 ewe lambs).

### 3.2. Period Around Midnight

**Figure 2:**
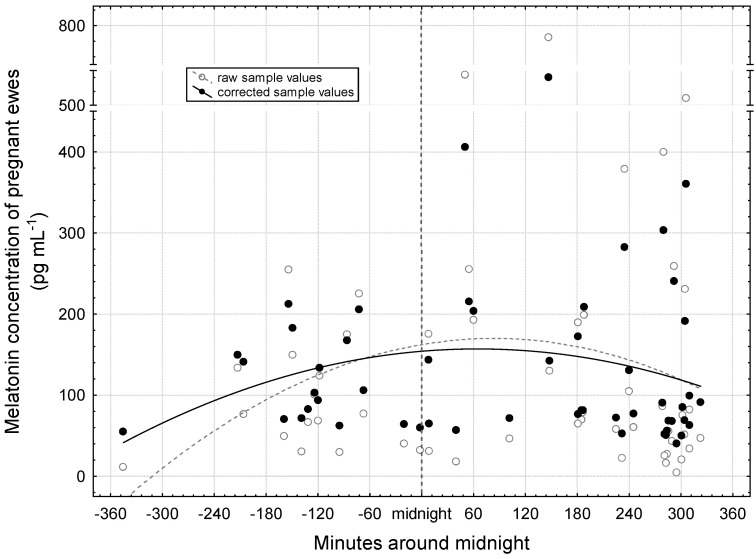
Distribution of nocturnal plasma melatonin concentration in pregnant ewes. The curve fitting on the raw sample and corrected sample values was performed with a quadratic function: raw sample values = 162.347 + 0.182 × x − 0.0011 × x^2^ and corrected sample values = 154.1456 + 0.0887 × x − 0.00007 × x^2^.

First, the nocturnal distribution of melatonin concentration is presented (Figure 2). A total of 53 samples evaluated during pregnancy were obtained from the 16 ewes (3.3 samples per individual on average). Both raw and corrected values have similar curves, showing an increase in the evening, a peak at night, and a decrease at dawn. For the correction, we took into account the gestational age and the days remaining until the winter solstice. This way we can evaluate the melatonin concentration exclusively in relation to the night hours. The peaks of the fitted quadratic curves can be located at around 60 min after midnight.

Using the quadratic curve function, adjusted values for the selected times of the night of the mid period (Table 1) were obtained. The number of dark hours on the night of 6 November to 7 November was 14 h and 17 min (sunset at 16:18 and sunrise at 06:35). The adjusted melatonin concentration was 68.8 pg mL^−1^ at 6:00 o’clock p.m. in the afternoon on 6 November. The values at 1:00 and 6:00 o’clock a.m. on 7 November were much higher (159.3 and 122.4 pg mL^−1^, respectively). Despite the deviations that could be considered essential, there was no significant difference between the values (*p* = 0.085).

### 3.3. Period Between Autumnal Equinox and Winter Solstice

**Figure 3:**
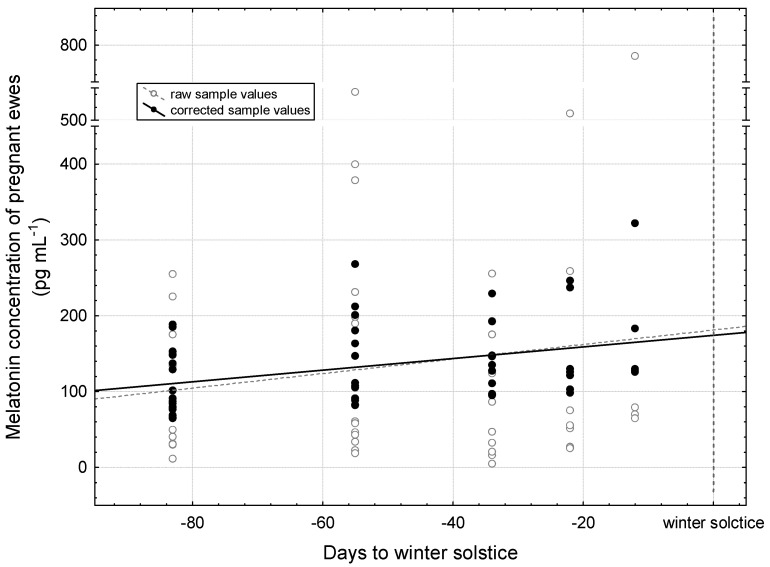
Distribution of plasma melatonin concentration by sampling from autumnal equinox until winter solstice in pregnant ewes. The curve fitting on the raw sample and corrected sample values was performed with a linear function: raw sample values = 181.2387 + 0.9568 × x; r = 0.1586; *p* = 0.257 and corrected sample values = 174.2618 + 0.7662 × x; r = 0.3459; *p* = 0.011.

During the examined period, raw values and corrected values showed an increase in melatonin concentration similarly to each other (Figure 3). Here, the adjusted values were freed from the effects of gestational age and sampling time (minutes around midnight). Linear curve fittings were used to demonstrate the trend which was significant on the corrected values (*p* = 0.011).

Table 2 presents, simultaneously, the melatonin concentration occurring at midnight and on specific days until winter solstice. The estimation was performed using linear function fitting. For the calculation of the adjusted values, observations made close to the given specific days were used. The lowest melatonin concentration is characteristic at midnight of the autumn equinox (127.5 pg mL^−1^). The highest concentration significantly differs from this at midnight of the winter solstice (188.3 pg mL^−1^, *p* < 0.001). At midnight of the mid time, a value between these two is formed (158.1 pg mL^−1^), with no statistically justified deviation from either.

### 3.4. Period of Gestation

**Figure 4:**
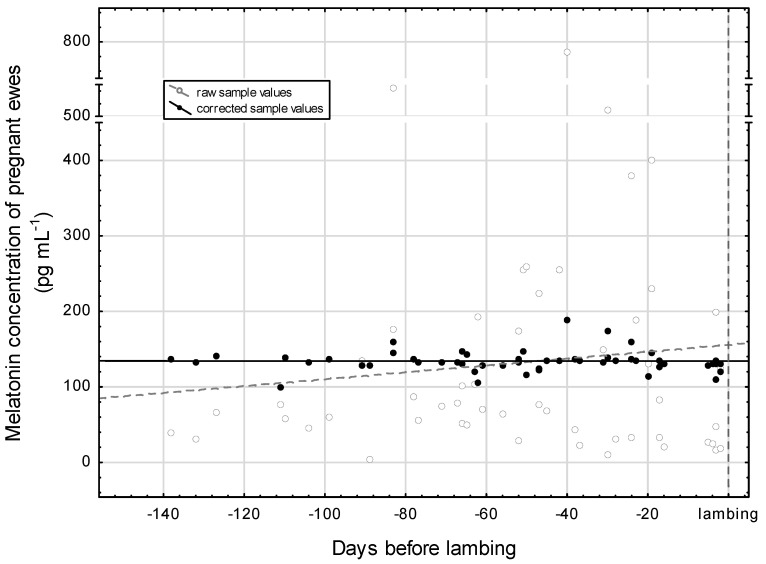
Distribution of plasma melatonin concentration during gestation in pregnant ewes. The curve fitting on the raw sample and corrected sample values was performed with a linear function: raw sample values = 155.7166 + 0.4549 × x; r = 0.1079; *p* = 0.442 and corrected sample values = 134.3457 − 0.0001 × x; r = −0.0003; *p* = 0.998.

In Figure 4, the distribution of raw sample values by the days to lambing are presented. The cloud of observations would suggest a gestational-age-dependent increase in the plasma melatonin concentration, but according to the statistics of the fitted linear function, this is not proven (*p* = 0.442). With regard to the corrected sample values, Figure 4 demonstrates a straight horizontal pattern. The correction was achieved by eliminating the effects of the minutes around midnight and the days to winter solstice. The statistical control proved that there is no gestational-age-related significant modification in the blood plasma melatonin concentration (*p* = 0.998). The average of the corrected sample values is around 134 pg mL^−1^ which is based on the first constant of the linear equation. In addition, the days to lambing in the study presented on the axis X testifies that the blood sampling practically cover the whole gestation period of the ewes.

Table 3 shows the plasma melatonin concentrations adjusted for midnight and specific days of the gestation. The specific days were chosen to explore the gestation at three different moments in time: d −98, −49, and −7 (which correspond to weeks 14, 7, and 1 before lambing, respectively). For the calculation of the adjusted values, we used observations made close to the given times. For example, there were 13 observations found close to d −98.

In our study, we found that the average of the plasma melatonin concentration was around 162 pg mL^−1^ at midnight. This is the reason why this value of 162.4 pg mL^−1^ is higher than the previously mentioned 134 pg mL^−1^. The melatonin concentrations by specific days before lambing do not differ greatly from each other (*p* = 0.783), which confirms our former result.

### 3.5. Period After Lambing

**Figure 5:**
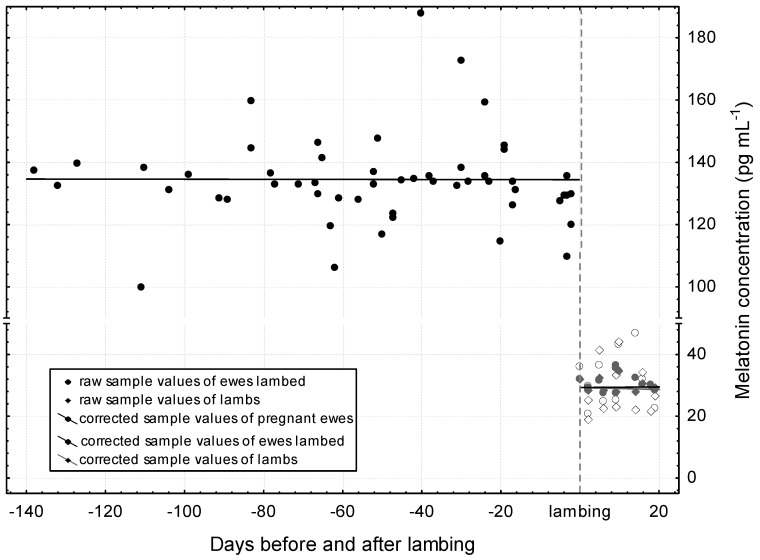
Distribution of plasma melatonin concentration before and after lambing. The curve fitting on the raw sample and corrected sample values was performed with a linear function: corrected sample values of ewes lambed = 30.162 + 0.0007 × x; r = 0.0019; *p* = 0.995 and corrected sample values of lambs = 30.2655−0.0291 × x; r = −0.0942; *p* = 0.771.

The nocturnal melatonin concentration after lambing is presented in Figure 5. By the end of the study period, 12 ewes had lambed. The figure shows the raw and corrected values of 12 dam–progeny pairs (one sample per individual and one randomly selected sample from one of the litter mates), supplemented by the corrected values of the pregnant dams before lambing (formerly shown in Figure 4). For the correction, we also excluded the effects of the minutes around midnight and the days leading up to the winter solstice.

Both raw and corrected values have similar straight curves and high *p*-values (0.995 and 0.771, respectively) in a linear regression model. The number of days since lambing has no influence on melatonin production. The concentration of melatonin produced after lambing is substantially lower than that before lambing and can be taken as 30 pg mL^−1^. The lambs have levels very similar to their mothers.

The melatonin concentration adjusted for midnight just 2 days before lambing (d −2) was 160.2 pg mL^−1^ (Table 4). Melatonin values were intentionally not corrected for the moment of lambing, as no samples were taken at this time. From this, the melatonin concentration adjusted for midnight just 1 day after lambing (d 1) in mothers and their lambs differed significantly (*p* < 0.001) uniformly (36.18 and 35.92 pg mL^−1^, respectively). In any case, a sample was taken from one mother–lamb pair on the night of lambing (d 0) (see Figure 5). The explanation for this is that we want to clearly separate the moment of lambing from the period preceding and following it. Low melatonin values in the postpartum period showed a smaller, one-fifth increase at midnight than in gestation period (approximately 6 vs. 28 pg mL^−1^).

## 4. Discussion

Regarding the pregnancy rate, it can be stated that soon after the opening of the harem, most of the ewes became pregnant, one after the other. In these animals, thirteen hours of continuous darkness was sufficient for the resumption of ovulatory cyclicity, which is in agreement with previous findings [11]. The second wave of pregnancy included four individuals that maintained seasonality, which conceived in late August and September, probably due to the cooler environment and the increase in the number of dark hours. It is known from the history of the breed that shepherds sometimes preferred to have their Tsigai flocks lamb by the end of the year [31].

According to Bittman et al. (1983), the produced melatonin enters the blood circulation with a pronounced day–night rhythm, characterized by low or undetectable concentrations (<10 pg/mL^−1^) during the day, which rise to levels of 200–400 pg/mL^−1^ at night [32]. In this study, we found the previously observed nightly change. The peak of melatonin concentration occurred after midnight. In our case, the *p*-value close to significant indicates that melatonin was already intensively produced by 6:00 p.m., and this increased only to a lesser extent during the night and persisted until dawn (6:00 a.m.). This is due to the autumn and early winter study period, when the number of daylight hours is less than 12 h. In a study conducted on the winter solstice, Carcangiu et al. (2013) detected the peak of about 150 pg/mL^−1^ at 8:00 p.m. in Sarda sheep (located in Sardinia, 39° N latitude) [33]. Then, they observed a persistence of melatonin concentration until 6:00 a.m. Additionally, they discovered that the melatonin hormone synthesis decreases with the age of the sheep.

Regarding the circannual variation, our results can be integrated with previous results obtained in the northern hemisphere [34]. During the period between the autumnal equinox and the winter solstice that we examined, nocturnal melatonin concentration showed a continuous and significant increase. The difference between the designated times is even greater if they are located further apart. The mean melatonin concentration of Ile-de-France ewes (in France, 45° N) was significantly lower in June (328.36 pg mL^−1^) than in December (400.46 pg mL^−1^) [35]. However, these figures are higher than ours. In the southern hemisphere, Coelho et al. (2006) measured in juvenile females in Brazil (22° S and 47° W) an average of 215.61 autumnal and 235.08 pg mL^−1^ winter melatonin concentration at 4:00 a.m. [36].

The natural plasma melatonin concentration in pregnant Tsigai ewes was successfully determined during the gestation period. In a previous study, investigated by Rollag et al. (1976), the nocturnal melatonin concentration showed a range of 100–300 pg mL^−1^ [37], which corresponds to this study, where the average of the corrected sample values is around 134 pg mL^−1^, that is, within the same range.

The 162 pg mL^−1^ melatonin concentration may be considered characteristic for midnights during fall (from the autumnal equinox until the winter solstice). Since there was no significant difference in the midnight melatonin concentration at different stages of gestation, we can state that the melatonin concentration of the ewes did not change according to the gestational age. We intentionally did not adjust melatonin values for the moment of lambing, as we did not collect samples at that time. Since blood samples were taken before and after parturition, melatonin concentrations were not measured precisely at the moment of birth. This gap leaves uncertainty about the immediate hormonal changes that occur during lambing. It is not known whether the gestational melatonin concentration remains at lambing or increases. However, the research at this time is a gap-filling study.

Our observation confirms the study performed in 13 Ile-de-France ewes, where Zarazaga et al. (1997) concluded that “melatonin secretion is unaffected by the stage of pregnancy” [26]. In a study conducted in Hungarian country donkeys, it was likewise shown that the melatonin concentration did not change significantly with advancing gestation [38]. Szczesna et al. (2018) investigated Polish Longwool ewes (synchronized and mated on October, northern hemisphere) throughout the entire gestation period (from day 30 to day 135) and collected samples every 15 days, after sunset [39]. They found that plasma melatonin concentrations significantly increased until day 60 of gestation (peaking at about 50 pg/mL, *p* < 0.05), and then decreased (to about 15 pg/mL, *p* < 0.05). In their study, the peak of melatonin coincided with the winter solstice, but they did not separate the effect of the number of daylight hours from the number of gestational days. Thus, the change in melatonin concentration is more likely to coincide with the relevant part of the circannual change. We deliberately made corrections for the gestational age and time remaining until the winter solstice in our analysis.

A research performed in humans, however, concluded that maternal melatonin and its metabolite, called 6-hydroxymelatonin sulfate (6-OHMS), showed much higher levels as the pregnancy of the women continued towards delivery; this means that the development of melatonin during pregnancy is different in humans compared to sheep [25]. Periodic monitoring of gestation in sheep has already been carried out. McMillen and Walker (1991) assessed plasma melatonin concentrations in late-gestational (120–138 days of gestation) catheterized mothers (cross-bred ewes, southern hemisphere, late spring and summer) and their catheterized fetuses [40]. During their study, the ewes had a mean highest nocturnal melatonin concentration (at 05:00 h) of 319 ± 58 pmol/L, while that of their fetuses (at 07:00 h) was 187 ± 16 pmol/L. This repeatedly demonstrated that melatonin is transferred from the sheep placenta to the fetus in the maternal–fetal direction [41], so that fetal melatonin depends on maternal melatonin production and its concentration follows the maternal concentration in accordance with darkness. A temporal analysis of plasma melatonin was carried out by Suarez-Trujillo et al. (2022) in periparturient dairy cows [42]. They stated that the daily mesor melatonin concentration before calving (d −35, −21, and −2), on the day of calving, and after calving (d 2, 5, 9, 15, and 22 postparturition) remained at about 3.5 ng/mL, without differences (*p* > 0.05). At the same time, they observed strong circadian rhythms in it.

However, similar to our results, the concentration decreased after the women had given birth and the placenta had been removed, from which point their level of melatonin would gradually drop to that of non-pregnant women. Because increased levels of 6-OHMS were found in the tissue of the placenta, this indicates that the placenta is a major source of maternal melatonin, as well as the source of an increased circulation of melatonin [25].

In this study, we first successfully established that melatonin concentrations in ewes decreased by a quarter, to 30 pg mL^−1^, immediately after parturition. Furthermore, this level was maintained for the first three weeks of the early puerperal period. We also found that the melatonin concentration of new-born lambs is identical to the reduced concentration of the mother one day after birth. The low values of the lamb and the mother persist for the first three weeks of the suckling period. Our present work confirmed the fact that new-born lambs do not yet have their own melatonin production [43], thus following the maternal level. The maternal melatonin, which transcends the placental barrier, is transferred to the fetus in utero via placental circulation, then to newborns via the milk [44]. This way, the fetus has the bonus of access to melatonin from its mother, even though the fetal pineal gland does not secrete melatonin. In the fetus, maternal melatonin appears to be an important factor for an early customization of the fetus to the circadian rhythms [45].

In our study, it was also confirmed that both of them have a non-pronounced diurnal nocturnal rhythm. Huchton (1984) correlated the melatonin profile in lambs with the maternal profile from day 5 postpartum [46]. He found detectable levels of nocturnal melatonin in the lambs (≤100 pg mL^−1^ on average) prolonged for eight weeks, at the same time in the adults, melatonin is released in a pulsatile manner in a higher range (>200 pg mL^−1^ on average). He also concluded that the endogenous melatonin rhythm of the mother did not directly influence the postnatal melatonin rhythm of the suckled lamb. For him, the identical melatonin concentrations in suckled and non-suckled lambs confirmed endogenous melatonin production starting from birth. Seron-Ferre et al. (2017) stated that the 5-days-old lambs have a low (about 30 pg mL^−1^ at the three clock times: 08:00, 14:00, and 20:00), steady plasma melatonin concentration for three weeks during the autumn–winter interval in the southern hemisphere [47]. According to them, a low constant postnatal level plays a role in early adrenal gland and heart neonatal adaptation. Nowak et al. (1990) also showed similarly low concentrations and a lack of circadian variation (in dark, 39.7 and in light, 39.5 pmol/L) in lambs younger than three weeks of age [48]. Between 3 and 4 weeks of age, they detected the onset of a diurnal rhythm in plasma melatonin concentrations (in dark, 164.1 and in light, 26.2 pmol/L; *p* < 0.001). Further on, they observed that these increased significantly by 6 weeks after birth and remained by 10 weeks of age, end of examined period [48]. In male juvenile animals (aged 3–9 months, West of Scotland, 55° N) in the study by Wyse et al. (2018), the plasma melatonin levels were closely related to day length in both winter and summer [49].

Antagonism between melatonin and prolactin (PRL) may be a plausible explanation for the sudden decrease in the maternal blood melatonin level. PRL is a hormone responsible for starting off and keeping up the lactation [50]. In a later phase of lactation, Molik et al. (2013) observed that melatonin has a negative influence on the secretion of prolactin via direct impact on pituitary cells (and of the growth-hormone (GH) as well) [51]. In their study, as usual, mothers lactated during the spring and summer, under a naturally increasing photoperiod, and melatonin concentrations were lower. Our analysis revealed that maternal melatonin production is also low before the winter solstice, when the photoperiod is at its lowest. In nursing ewes, GH secretion significantly increases during intense suckling, especially during the first 10–30 min of suckling [52]. Schmitt et al. (2014) investigated salivary melatonin concentration on ewes on day 14 postpartum [53]. They explained the lack of daily melatonin cyclicity caused by the stress that the animals were exposed to during blood sampling, based on former experiences [54]. They also measured the salivary cortisol (CORT) concentration, and it is believed that its constant values are attributed to the evenness of the suckling. According to Walker et al. (1992), the secretion of CORT is a response to the sucking stimulus [55]. In the small-sized Tsigai flock we studied, pregnant ewes lived together in the same environment as suckler ewes and their lambs. The hypothalamic–pituitary–adrenal (HPA) axis is the body’s response to stress. During stress, the hypothalamus of mammals produces corticotrophin-releasing hormone (CHR), which increases the level of adrenocorticotropic hormone (ACTH) in the pituitary gland [56]. Subsequently, the level of CORT increases [57]. The function of cortisol is to help the body cope with stress, regulate metabolism, and modulate immune system responses [58]. The diurnal course of CORT is usually opposite to that of melatonin, with higher levels during the day. Melatonin inhibits the secretion of CHR [59]. This leads to a decrease in ACTH, which in turn reduces CORT levels. The reduction in cortisol levels by melatonin does not interfere with stress responses and potentially supports the maintenance of homeostasis by preventing excessive cortisol secretion, which may be detrimental in the long term [60]. Human observations have shown that fasting serum cortisol and cortisol pulse frequency increase at the end of pregnancy [61]. It is conceivable that cortisol and melatonin increase together, so that, in this case, the increase in the melatonin concentration at the end of pregnancy is also due to the increase in cortisol levels. In order to clarify this causal relationship in sheep, such studies are needed.

We demonstrated moderate diurnal cyclicity in pregnant ewes, but not in lactating ewes and their lambs. We suggest that the decrease in melatonin may be explained by the maintenance of wakefulness in both the mother and the newborn, especially during the night. Oxytocin plays a significant role in the formation of the bond between the two [62,63]. Protection and breastfeeding in the dark are developed on a special relationship that develops after birth, based on olfactory cues [64], since their recognition of each other based on vision and sound is less reliable [65].

In non-human primate fetuses, melatonin may help limit the production of cortisol, a stress hormone produced by the suprarenal gland. All this indicates that melatonin contributed to better placental development in general [66] and may influence important fetal functions needed for neonatal adaptation to life outside the womb [67]. Melatonin has an antioxidant effect to avoid hypoxic brain damage in neonates during parturition; despite this, Beñaldo et al. (2019) received a low daytime concentration of melatonin (<5 pg mL^−1^ at 14:00) in lambs as old as two weeks under experimental housing conditions [68].

In general, it can be stated that correction leads to more reliable results and at the same time favorably reduces the variability of the data set. A possible reason for the differing results in humans and sheep may be due to more carefully performed statistical data processing in sheep, involving more background information.

## 5. Limitations

This study focused exclusively on Tsigai ewes, an old heritage breed, so the general applicability of its findings is limited. Since melatonin secretion and reproductive physiology can vary between sheep breeds, the results may not be directly applicable to other breeds with different genetic backgrounds. Especially in cultured breeds, where selection pressures are much stronger, physiological changes may follow different patterns than genetic changes.

## 6. Conclusions

Our present study showed that there was no significant change in the concentration of plasma melatonin during the gestation of the Tsigai ewes subjected to our investigation in Middle Europe. It can be stated that correction leads to more reliable results and at the same time favorably reduces the variability of the dataset. We consider our statistical approach convincing. It led to acceptable results while allowing for a reduction in the number of animals required for sampling. Our results confirmed the previous single observation of melatonin levels in pregnant sheep. A possible reason for the differing results in humans and sheep may be due to more carefully performed statistical data processing in sheep, involving more background information. Or the urbanized human lifestyle is not so similar to natural processes and environments, with respect to light–dark, climate, and noise. It was also confirmed that both mothers and lambs have a non-pronounced diurnal nocturnal rhythm. Both have low melatonin concentrations after birth. We consider it necessary to determine the melatonin concentration at lambing and immediately around the time of lambing.

## Figures and Tables

**Table 1:** The melatonin concentration adjusted for specific day time at mid period.

Specific Day Time *p* = 0.085	No. of Observations Considered ^†^	Melatonin Concentration Mean, pg mL^−1^	SEM *
Mid period (6–7 November 2023):			
Afternoon 6:00 p.m.	11	68.8	15.71
Melatonin top after midnight 1:00 a.m.	21	159.3	26.20
Morning 6:00 a.m.	30	122.4	20.60

† Those pregnant ewes whose sampling was closest to the designated day times. Minute of sampling <−100, >−150 and <150, and >100, respectively. * Standard error of mean.

**Table 2:** The melatonin concentration adjusted for midnight and specific days until winter solstice.

Specific Days *p* < 0.001	No. of Observations Considered ^†^	Melatonin Concentration Mean, pg mL^−1^	SEM *
Autumnal equinox (23–24 September 2023)	29	127.5 ^a^	9.13
Mid time (6–7 November 2023)	25	158.1 ^ab^	10.24
Winter solstice (21–22 December 2023)	22	188.3 ^b^	12.44

† Those pregnant ewes whose sampling was closest to the specific days. Days of sampling <−50, >−50 and <−30, and >−40, respectively. * Standard error of mean. a, b: Different superscript letters differ significantly (*p* < 0.001).

**Table 3:** The melatonin concentration adjusted for midnight according to specific days of gestation.

Days to Lambing *p* = 0.783	No. of Observations Considered ^†^	Melatonin Concentration Mean, pg mL^−1^	SEM *
98 days before lambing	13	162.6	3.66
49 days before lambing	22	163.7	3.73
7 days before lambing	18	160.5	2.67

† Those pregnant ewes whose samplings took place close to the given times. * Standard error of mean.

**Table 4:** The melatonin concentration adjusted for before and after lambing and for midnight.

Specific Days *p* < 0.001	No. of Observations Considered ^†^	Melatonin Concentration Mean, pg mL^−1^	SEM *
Adjusted for 2 days before lambing in pregnant ewes	22	160.2 ^a^	2.85
Adjusted for 1 day after lambing in ewes lambed	12	36.18 ^b^	0.634
Adjusted for 1 day after birth in lambs	12	35.92 ^b^	0.578

† Pregnant ewes 40 days before lambing, all ewes lambed, and all their lambs. * Standard error of mean. a, b: Different superscript letters differ significantly (*p* < 0.001).

## Data Availability

The data that support the findings of this study are available from the corresponding author upon reasonable request.

## Supplementary Materials

The following supporting information can be downloaded at: https://www.mdpi.com/article/10.3390/vetsci12040336/s1, Figure S1: Timeline for experimental procedure; Figure S2: The standard curve of the analysis.
