# Supplementary figures and images for: Determination of Natural Blood Plasma Melatonin Concentration of Tsigai Ewes Characteristic for Gestation and Early Postpartum Period Between Autumnal Equinox and Winter Solstice
Source: Vet Sci. 2025 Apr 5;12(4):336. doi: 10.3390/vetsci12040336 (PMC12031133; doi:10.3390/vetsci12040336)

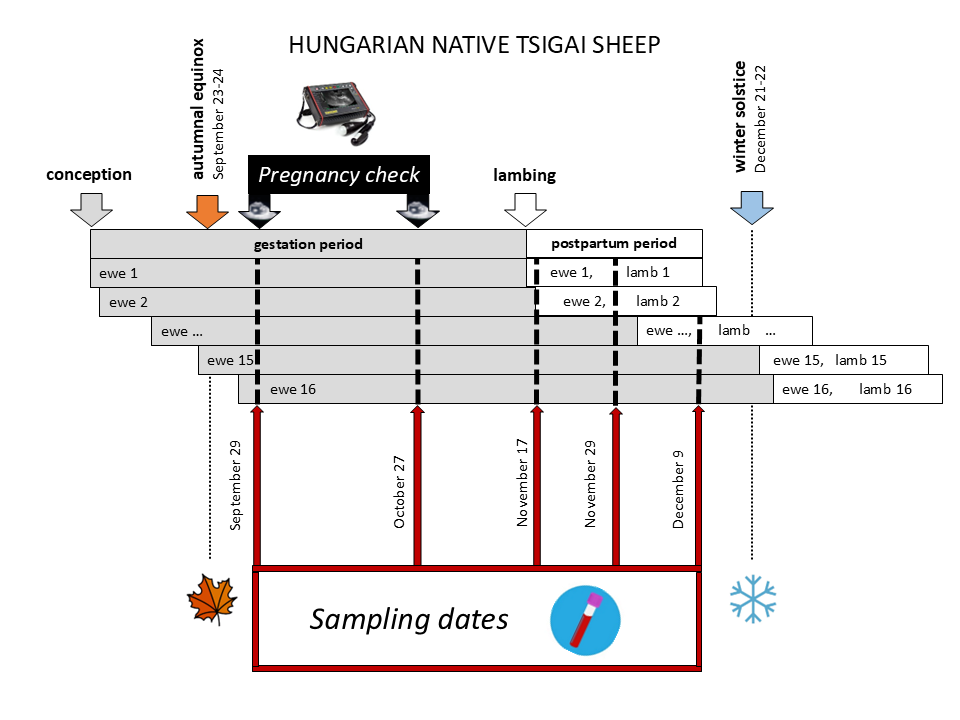

Supplement: Supplementary file 1 [file vetsci-12-00336-s001.zip › Figure S1 updated.png]

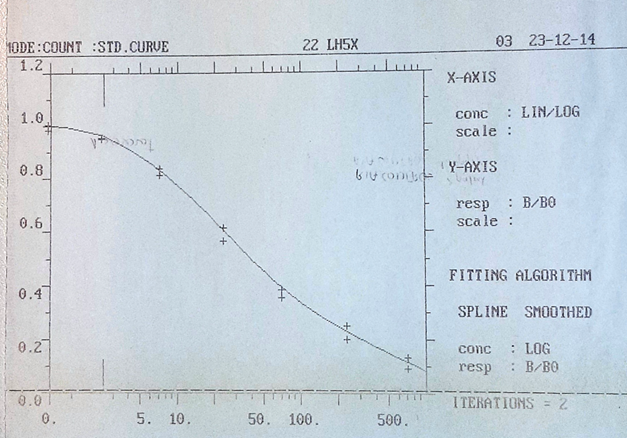

Supplement: Supplementary file 1 [file vetsci-12-00336-s001.zip › Figure S2.png]
